# Paediatric musculoskeletal matters (pmm) – collaborative development of an online evidence based interactive learning tool and information resource for education in paediatric musculoskeletal medicine

**DOI:** 10.1186/s12969-015-0062-4

**Published:** 2016-01-05

**Authors:** Nicola Smith, Tim Rapley, Sharmila Jandial, Christine English, Barbara Davies, Ruth Wyllie, Helen E. Foster

**Affiliations:** Paediatric Rheumatology, Musculoskeletal Research Group, Institute Cellular Medicine, Newcastle University, Newcastle Upon Tyne, UK; Institute of Health and Society, Newcastle University, Newcastle Upon Tyne, UK; Great North Children’s Hospital, Newcastle upon Tyne Hospitals NHS Trust, Newcastle Upon Tyne, UK; Department of Public Health and Wellbeing, Northumbria University, Newcastle Upon Tyne, UK

**Keywords:** Education, Online resource, e-learning, Clinical skills, pGALS, pREMS, Normal musculoskeletal development, Musculoskeletal medicine, Children and Young People, Orthopaedics, Rheumatology, Neuromuscular medicine, pmm

## Abstract

**Background:**

We describe the collaborative development of an evidence based, free online resource namely ‘paediatric musculoskeletal matters’ (pmm). This resource was developed with the aim of reaching a wide range of health professionals to increase awareness, knowledge and skills within paediatric musculoskeletal medicine, thereby facilitating early diagnosis and referral to specialist care.

**Methods:**

Engagement with stakeholder groups (primary care, paediatrics, musculoskeletal specialties and medical students) informed the essential ‘core’ learning outcomes to derive content of pmm. Representatives from stakeholder groups, social science and web development experts transformed the learning outcomes into a suitable framework. Target audience representatives reviewed the framework and their opinion was gathered using an online survey (*n* = 74) and focus groups (*n* = 2). Experts in paediatric musculoskeletal medicine peer reviewed the content and design.

**Results:**

User preferences informed design with mobile, tablet and web compatible versions to facilitate access, various media and formats to engage users and the content presented in module format (i.e. Clinical assessment, Investigations and management, Limping child, Joint pain by site, Swollen joint(s) and Resources).

**Conclusions:**

We propose that our collaborative and evidence-based approach has ensured that pmm is user-friendly, with readily accessible, suitable content, and will help to improve access to paediatric musculoskeletal medicine education. The content is evidence-based with the design and functionality of pmm to facilitate optimal and ‘real life’ access to information. pmm is targeted at medical students and the primary care environment although messages are transferable to all health care professionals involved in the care of children and young people.

## Background

Musculoskeletal (MSK) complaints in children and young people (CYP) are common with a wide spectrum of causes [1–4]. In many cases the cause of the symptoms is benign, self-limiting or trauma related but the differential diagnosis is broad and MSK symptoms can be presenting features of serious and potentially life threatening illnesses such as malignancy, vasculitis, sepsis, non-accidental injury and also chronic diseases such as muscle disease, arthritis or neurodisability. Delay in diagnosis and access to care are well reported in childhood diseases that often present with MSK features including cancer, muscular dystrophy and juvenile idiopathic arthritis (JIA) [4–7]. The reasons for delay are multifactorial [8] including complex pathways, and CYP with MSK concerns invariably present to primary care or various secondary care specialties (usually general paediatrics, orthopaedics or emergency care) [9]; many such doctors report a lack of awareness about MSK disease in CYP, and in particular about arthritis in CYP. Likewise, evidence suggests that many doctors lack confidence and competence in their paediatric MSK clinical skills [5–7, 10] stemming from a lack of MSK teaching at undergraduate [11] and postgraduate level [12–15]. Such unmet need is also observed in nurses who may encounter CYP in their clinical practice [16, 17]. Patients and families of CYP with chronic MSK disease report that raising awareness, earlier diagnosis and referral for specialist care are amongst their top priorities to improve clinical outcomes [personal communication Sharon Douglas UK Paediatric Rheumatology Clinical Studies Group (CSG), [18].

We have previously developed novel examination tools to aid joint examination in CYP; paediatric Gait Arms Legs Spine (pGALS) [19] and paediatric Regional Examination of the Musculoskeletal System (pREMS) [20]; and core MSK learning outcomes for primary care doctors [21] and medical students [22]. pGALS is widely taught in medical schools in the UK [23] and internationally. Since publication in 2011, pREMS is increasingly taught albeit targeted at postgraduate training. The interpretation of pGALS and pREMS needs knowledge and context to optimise their utility in clinical practice. This manuscript describes the process to develop Paediatric Musculoskeletal Matters (pmm) – www.pmmonline.org - as an online resource designed to host resources to facilitate learning of pGALS, pREMS and core learning needs. pmm was designed originally to target medical students and primary care doctors, although the content is relevant to paediatricians and other clinicians involved in the care of CYP.

## Methods

### Collaborative design and development

The project team included academics and clinicians from Newcastle and Northumbria Universities and the Great North Children’s Hospital, Newcastle Hospitals National Health Service (NHS) Foundation Trust. The team encompassed paediatric rheumatology, adult rheumatology, paediatric orthopaedics, paediatric neuromuscular medicine, general practice, general paediatrics, medical sociology, education and health psychology. Clinicians in the project team included doctors, nurses and allied health professionals.

pmm was designed and built in collaboration with key user stakeholders (including medical students, nurses, primary care and paediatrics) along with industry partners specialising in web and media design. Extensive user testing and feedback was gathered at each stage of development with the design and development being iteratively updated. This process is based on collaborative design principles from research and practice in human-computer interaction and computer-supported cooperative work; often referred to as cooperative design, participatory design or co-design [24]. It was deemed important that pmm was designed with potential users to ensure engagement with the target audience from the start and to enable the resource to meet their required needs rather than merely designing a resource for them. We undertook three phases of work;

i. Design and development phase: An anonymised web-based e-survey. Participants (*n* = 74) representing the target audience of pmm included medical students (*n* = 17), primary care doctors (*n* = 1), primary care trainee doctors (*n* = 11), primary care doctor trainers (*n* = 6), paediatric trainees (*n* = 7), primary care nurses (*n* = 2), paediatric nurses (*n* = 14), student nurses (*n* = 11) and nurses working in other health care environments (*n* = 5). Participants were asked about current use of online technologies in relation to clinical practice and learning, seeking and access to information online, websites that they frequently use (and why), factors influencing online use and any problems encountered with using reference and e-learning sites. The survey findings were further explored through two user focus groups (*n* = 15 participants in total, and with a spread of users at different levels of training). Feedback was recorded with notes and not audio-recorded, with comments anonymised in accordance with industry website development practice. Descriptive statistics analysed the survey with user group data analysed following standard procedures for qualitative analysis, including open and focused coding, constant comparison and deviant case analysis [25].
ii. Usability testing: The project team and representatives from the stakeholder groups were asked to view the proposed site and provide feedback. Further users viewed a wireframe of the proposed web or mobile version of the design. Potential users were invited to complete four simple tasks to encourage exploration of the site and then asked to provide detail on their experience through the completion of a web-based survey.
iii. Content Development: The framework for pmm was developed through engagement with stakeholder groups. The content, based on evidence of need [21, 22] established MSK themes (appropriate for primary care and graduating medical students), and was written by the project team with additional authors from primary care with an interest in education and musculoskeletal medicine. The content was subjected to a structured content and governance approval process with final ‘sign off‘ by senior members of the clinical team (HF, SJ, RW). To improve the content validity, expert peer review from UK paediatric rheumatology, orthopaedics and neuromuscular medicine was conducted and paediatric rheumatologists from countries and health care systems outside the UK. Suggestions for additional content or amplification of details were welcomed and addressed in the content.

This process was completed in accordance with regular industry website development practice and had ethical approval (Newcastle University Ethics Committee).

## Results

### Initial design considerations

pmm needs to be an open access e-resource with ‘wide reach’ to stakeholders who are not specialists in musculoskeletal medicine. pmm needs to capture user access to the site (Google Analytics) including popular pages, search terms and feedback (e-survey) to facilitate further development. Regarding content and user functionality, five core themes emerged from the survey and focus groups and these are discussed below:

#### Theme 1: mobile/Web compatibility

pmm needs be compatible across all platforms and a “*mobile compatible version of the site should be designed in addition to a web-based version*”. Responders highlighted limitations associated with their experience of *“old and slow computers at work”* that “*could restrict access to the site”*, and suggested that, *“any resource developed should also be compatible with these older operating systems”*.

#### Theme 2: content design

pmm content needs to be concise and relevant; “*need to know essential information easily understood by the target audience”* and *“presented in sections with summaries and key points”*. User focus groups suggested key themes, topic areas and appropriate formats; these included content presented in ‘core’ clinical presentations (such as the limping child or knee pain), approaches to assessment and initial management, practical advice on when to be concerned (i.e. ‘red flags’), videos particularly in realistic situations/environments with practical demonstrations of key points or clinical skills. Users highlighted the importance of ‘being up to date’ and validity of the site as a trusted resource; citing links to key relevant websites (e.g. National Institute for Health and Care Excellence guidelines (www.NICE.org.uk), review articles and professional logos and endorsement (e.g. University, NHS Trust, professional organisations) were deemed important to optimise credibility.

#### Theme 3: effective navigational design

An effective search facility is important to optimise site navigation; *“time is of the essence – I need to quickly have a look…”, “must be quick so I can use between patients…”* and suggestions to optimise the search facility included: *“refining search criteria by information format (*e.g. *text, video or information sheet)”*; “*use of hyperlinks to guidelines or further information in the site”*; and *“menu bars and subject headings being in clear sections”*. Users described that they would “*use the site as both a quick information resource or reference within their clinical practice”* (e.g. in clinics, between patient encounters) and as a learning tool (e.g. study time or private time). Users need to explore the site in *“as few clicks”* as possible but want to bookmark pages and explore these later in private time.

#### Theme 4: aesthetic quality

Users suggested a *“slick”*, *“professional”* and *“modern”* look with *“clear fonts”*, *“obvious links”* and “*clear consistent backgrounds free from clutter”* to be utilised within the design without adverts or ‘pop ups’.

#### Theme 5: facilitating access

Users preferred the site to be *“free of charge”* and open; *“no need to register to access as much of the content as possible”*. Most users however appreciated that the sensitive nature of some content (e.g. patient videos) would require registration from a governance perspective. Registration was also deemed acceptable to enable bookmarking of pages, compilation of a personal dashboard (‘favourite/most useful’ pages) and a log of accessed pages.

Examples of websites with positive and negative design qualities informed pmm site design with wireframes (namely, screen prototypes) developed to explore the design further, particularly in relation to usability.

### Usability testing

Usability testing of the proposed pmm site along with web and mobile versions was conducted by project team and stakeholder representatives (*n* = 20); testing involved search tasks and opinion on format. Feedback was positive; *“Very easy to follow and navigate”, “Easy to find what you’re looking for”*; “*The layout of the content made logical sense and I liked that all the section headings were ‘exploded’ along the left hand side of the page”*. A full test website was then developed with further usability testing within the stakeholder group (*n* = 10) confirming the resource to be accessible, acceptable and user friendly.

### Development of core content

**Fig. 1:**
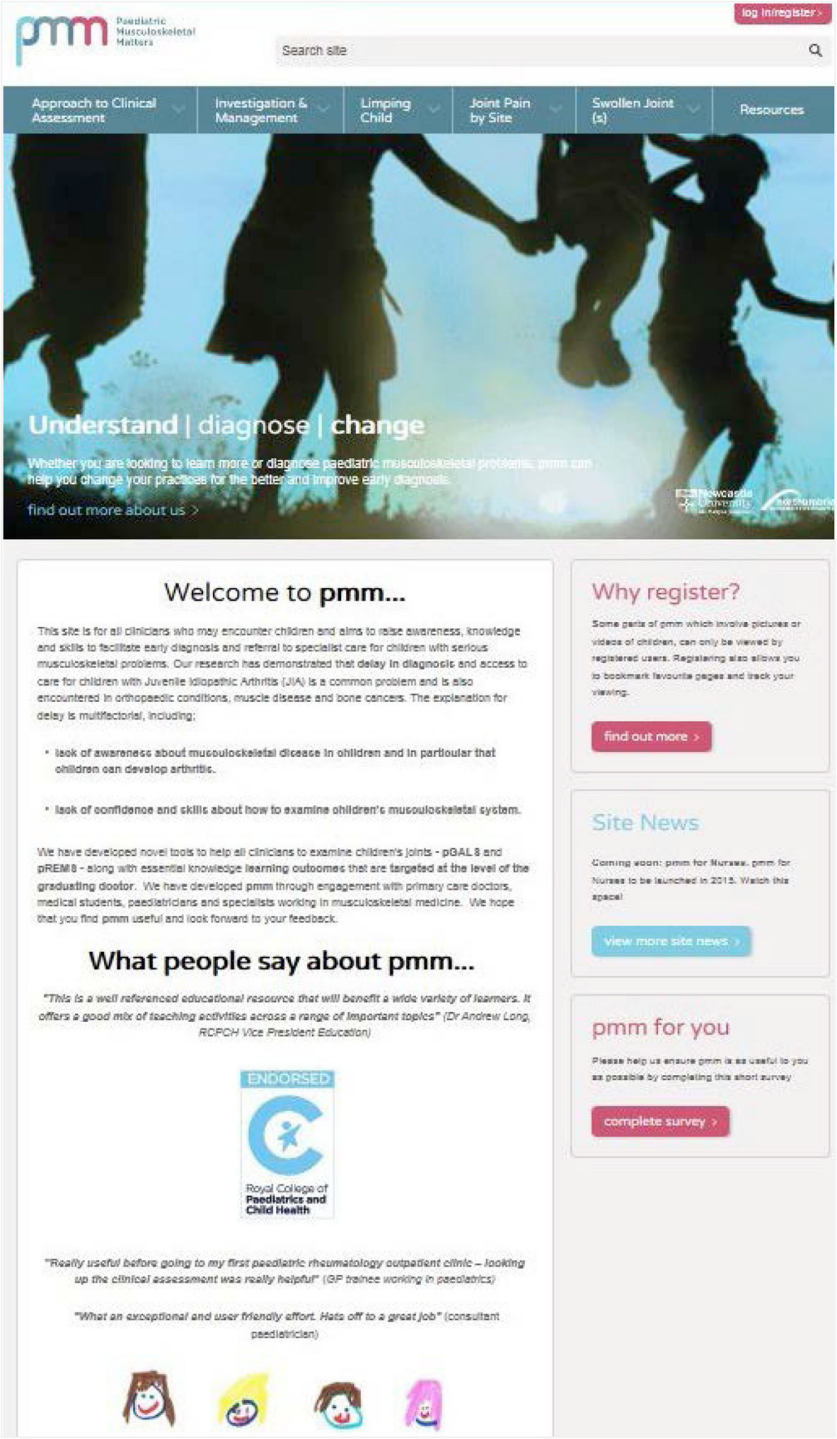
Screen shot of pmm homepage

The pmm content is presented in six modules - Approach to clinical assessment, Investigations and management, Limping child, Pain by site, Swollen joint(s), and Resources (see Fig. 1). Within each module, sub-modules present information in complementary ways (videos, images, red flags, referral guidance, cases, summary points [‘Top Tips’] and links to related pages in other modules and recommended websites, guidelines and key references within Resources). Videos to demonstrate pGALS, pREMS and illustrative clinical signs are available albeit through registered access. Each module contains essential information in short notes form and includes topics within rheumatology, orthopaedics, neuromuscular medicine and ‘red flag’ conditions (i.e. cancer, infection and non accidental injury).

pmm was launched in November 2014 and so far with reach to >100 countries across the world (see Table 1), >1000 registered users and > 60,000 ‘page hits’ (Data provided by Box Model Digital Media, 2015 - http://www.boxmodeldigital.com). The most popular pages have been within clinical assessment, namely; pGALS, pREMS, video demonstrations, normal MSK development and referral guidance. The videos of joint examination (pGALS and pREMS) have been very popular with paediatric trainees as part of their clinical skills training.

**Table 1:** Countries accessing site

Country (Top 20)	Sessions
1. United Kingdom	6849 (36.69 %)
2. United States	3097 (16.59 %)
3. India	1405 (7.53 %)
4. New Zealand	909 (5.14 %)
5. Australia	601 (3.22 %)
6. Canada	339 (1.82 %)
7. Brazil	309 (1.66 %)
8. Saudi Arabia	303 (1.62 %)
9. Ireland	245 (1.31 %)
10. Germany	204 (1.09 %)
11. Russia	196 (1.05 %)
12. China	191 (1.02 %)
13. Malaysia	172 (0.92 %)
14. Japan	161 (0.86 %)
15. South Africa	161 (0.86 %)
16. United Arab Emirates	145 (0.78 %)
17. Spain	134 (0.72 %)
18. Italy	126 (0.67 %)
19. South Korea	122 (0.65 %)
20. Netherlands	122 (0.65 %)
Other countries included:	
Egypt, France, Pakistan, Colombia, Turkey, Indonesia, Hungary, Hong Kong, Thailand, Greece, Mexico, Philippines, Sweden, Singapore, Israel, Taiwan, Czech Republic, Kenya, Poland, Portugal, Kuwait Argentina, Denmark, Lithuania, Oman, Iran, Norway, Belgium, Ukraine, Finland, Switzerland, Austria, Jordan, Barbados, Qatar, Romania, Iraq, Sudan, Sri Lanka, Vietnam, Nigeria, Bahrain, Bangladesh, Bulgaria, Serbia, Chili, Palestine, Venezuela, Croatia, Lebanon, Nepal, Papua New Guinea, Peru, Botswana, Costa Rican Ecuador, Ethiopia, Georgia, Latvia, Morocco, Slovakia, El Salvador, Libya, Macedonia, Malta, Puerto Rico, Brunei, Cyprus, Iceland, Myanmar, Mongolia Tunisia, Tanzania, Bosnia & Herzegovina, Estonia, Fiji, Grenada, Ghana, Cayman Islands, Bolivia, Gibraltar, St Kitts & Nevis, Kazakhstan, Luxembourg, Panama, Slovenia, Trinidad & Tobago, Zimbabwe, Armenia, Angola, Azerbaijan, Belarus, Dominican Republic, Guernsey, Jersey, Jamaica, Cambodia, Syria, Uruguay, Uzbekistan, Kosovo, Benin, Bhutan, Curacao, Dominica, Algeria, Gabon, Guinea, Honduras, Moldova, Mali, Mauritania, Mauritius, Mozambique, New Caledonia, Paraguay, Senegal, Somalia, Suriname, Tonga, Uganda, Yemen.	
Total Number of countries accessing site: 143	
Based on Google Analytic Data Collected 15/12/15	

Peer review of pmm was invited from UK professional organisations (e.g. British Society for Paediatric and Adolescent Rheumatology (BSPAR), Royal College Paediatrics and Child Health (RCPCH) and also international colleagues within Pediatric Rheumatology Society (PRES). Feedback has been very positive to support design, format and content of pmm. Peer review has suggested additional content to optimise relevance of pmm to an international audience (e.g. more information on differential diagnoses [such as rheumatic fever and mycobacterial disease] as well as further images to reflect ethnic diversity). In addition, translations of key pmm material into different languages has been requested (e.g. the pGALS maneuvers and instructions), and this work is in progress working with international colleagues as contributors.

## Discussion and conclusions

Our collaborative and evidence based approach to develop pmm has resulted in an open free e-resource, available using various technologies and targeting non-specialists in paediatric musculoskeletal medicine. This resource – paediatric musculoskeletal matters- pmm- we believe, is the first such resource to target these user groups. User engagement from the start aimed to ensure design and functionality to address their respective needs.

The content of pmm is evidence-based to address learning outcomes for medical students and primary care although the essential knowledge is important to paediatric trainees, general paediatricians and indeed all clinicians involved in the care of CYP. It is envisaged that pmm will be useful to members of the international paediatric rheumatology community who are engaged in the teaching of medical students and non-specialist clinicians. A parallel site – pmm for nurses - is in progress (launch due 2016). We envisage that pmm is an important step to inform a wide target audience, provide knowledge to improve assessment of CYP with MSK presentations and ultimately facilitate earlier diagnosis and referral to specialists.

We acknowledge limitations in our approach. The content of pmm is based on the learning outcomes with the target audience of medical students and primary care doctors working in the UK. The stakeholder representatives were from the UK and mainly recruited from the North of England. However the process of peer review and user feedback since the launch of pmm has involved a wider (UK and international) audience. Feedback confirmed the design and functionality of pmm to be appropriate and english remains the preferred language although translations of key pages (e.g. pGALS instructions) have been requested. Iterations of pmm after peer review have enabled further content to be added (e.g. neuromuscular medicine) and optimised the validity of the resource. pmm is relevant to a spectrum of users (other than medical students and primary care) and regarded as a foundation for further learning; paediatricians who wish to build on their knowledge are encouraged to pursue further training (e.g. EULAR online course in paediatric rheumatology - http://www.eular.org/edu_online_course_paediatric.cfm).

Peer review has also suggested further clinical images to reflect ethnic diversity and the spectrum of disease presentations in different parts of the world. pmm for India (September 2015) is an exemplar of partnership between the project team and local paediatricians to develop pmm for their health care context. Further ‘internationalisation’ of pmm is planned as well as a pmm app to aid access to information where Internet access is limited. Future work will focus on evaluating pmm in terms of user experience, real-time access and how pmm influences learning and decision making in the clinical context.

### Further information

The resource can be found at www.pmmonline.org.

## Abbreviations

CYP: children and young people
CSG: Clinical Studies Group
JIA: juvenile idiopathic arthritis
MSK: musculoskeletal
NHS: National Health Service
NICE: National Institute for Health and Care Excellence
pmm: paediatric musculoskeletal matters
pGALS: paediatric gait arms legs spine
pREMS: Paediatric Regional Examination of the Musculoskeletal System
